# Đakrông virus, a novel mobatvirus (*Hantaviridae*) harbored by the Stoliczka’s Asian trident bat (*Aselliscus stoliczkanus*) in Vietnam

**DOI:** 10.1038/s41598-019-46697-5

**Published:** 2019-07-15

**Authors:** Satoru Arai, Keita Aoki, Nguyễn Trường Sơn, Vương Tân Tú, Fuka Kikuchi, Gohta Kinoshita, Dai Fukui, Hoàng Trung Thành, Se Hun Gu, Yasuhiro Yoshikawa, Keiko Tanaka-Taya, Shigeru Morikawa, Richard Yanagihara, Kazunori Oishi

**Affiliations:** 1 Infectious Disease Surveillance Center, National Institute of Infectious Diseases, Tokyo, 162-8640 Japan; 2 Tokyo University of Science, Tokyo, 162-8601 Japan; 3 Institute of Ecology and Biological Resources, Vietnam Academy of Science and Technology, Hanoi, Vietnam; 4 Graduate University of Science and Technology, Vietnam Academy of Science and Technology, Hanoi, Vietnam; 5 Kyoto University Graduate School of Agriculture, Kyoto, 606-8502 Japan; 6 The University of Tokyo Hokkaido Forests, Graduate School of Agricultural and Life Sciences, The University of Tokyo, Furano, Hokkaido 079-1561 Japan; 7 Faculty of Biology, University of Science, Vietnam National University, Hanoi, Vietnam; 8 Pacific Center for Emerging Infectious Diseases Research, John A. Burns School of Medicine, University of Hawaii at Manoa, Honolulu, HI 96813 USA; 9 Chiba Institute of Science, Chiba, 388-0025 Japan; 10 Department of Veterinary Science, National Institute of Infectious Diseases, Tokyo, 162-8640 Japan

**Keywords:** Viral evolution, Viral reservoirs

## Abstract

The recent discovery of genetically distinct shrew- and mole-borne viruses belonging to the newly defined family *Hantaviridae* (order Bunyavirales) has spurred an extended search for hantaviruses in RNAlater®-preserved lung tissues from 215 bats (order Chiroptera) representing five families (Hipposideridae, Megadermatidae, Pteropodidae, Rhinolophidae and Vespertilionidae), collected in Vietnam during 2012 to 2014. A newly identified hantavirus, designated Đakrông virus (DKGV), was detected in one of two Stoliczka’s Asian trident bats (*Aselliscus stoliczkanus*), from Đakrông Nature Reserve in Quảng Trị Province. Using maximum-likelihood and Bayesian methods, phylogenetic trees based on the full-length S, M and L segments showed that DKGV occupied a basal position with other mobatviruses, suggesting that primordial hantaviruses may have been hosted by ancestral bats.

## Introduction

The long-standing consensus that hantaviruses are harbored exclusively by rodents has been disrupted by the discovery of distinct lineages of hantaviruses in shrews and moles of multiple species (order Eulipotyphla, families Soricidae and Talpidae) in Asia, Europe, Africa and North America^1,2^. Not surprisingly, bats (order Chiroptera, suborders Yangochiroptera and Yinpterochiroptera), by virtue of their phylogenetic relatedness to shrews and moles and other placental mammals within the superorder Laurasiatheria^3,4^, have also been shown to harbor hantaviruses^1,2^. Subsequently, based on phylogenetic analysis of the full-length S- and M-genomic segments, members of the genus *Hantavirus* (formerly family *Bunyaviridae*) have been reclassified into four newly defined genera (*Loanvirus*, *Mobatvirus, Orthohantavirus* and *Thottimvirus*) within a new virus family, designated *Hantaviridae*^5,6^.

All rodent-borne hantaviruses, as well as nearly all newfound hantaviruses hosted by shrews and moles, belong to the genus *Orthohantavirus*^6^. By contrast, bat-borne hantaviruses have been assigned to the *Loanvirus* and *Mobatvirus* genera. To date, bat-borne loanviruses include Mouyassué virus (MOYV) in the banana pipistrelle (*Neoromicia nanus*) from Côte d’Ivoire^7^ and in the cape serotine (*Neoromicia capensis*) from Ethiopia^8^, Magboi virus (MGBV) in the hairy slit-faced bat (*Nycteris hispida*) from Sierra Leone^9^, Huángpí virus (HUPV) in the Japanese house bat (*Pipistrellus abramus*) from China^10^, Lóngquán virus (LQUV) in the Chinese horseshoe bat (*Rhinolophus sinicus*), Formosan lesser horseshoe bat (*Rhinolophus monoceros*) and intermediate horseshoe bat (*Rhinolophus affinis*) from China^10^, and Brno virus (BRNV) in the common noctule (*Nyctalus noctula*) from the Czech Republic^11^.

Mobatviruses include Xuân Sơn virus (XSV) in the Pomona roundleaf bat (*Hipposideros pomona*) from Vietnam^12,13^, Láibīn virus (LAIV) in the black-bearded tomb bat (*Taphozous melanopogon*) from China^14^, Makokou virus (MAKV) in the Noack’s roundleaf bat (*Hipposideros ruber*) from Gabon^15^, and Quezon virus (QZNV) in the Geoffroy’s rousette (*Rousettus amplexicaudatus*) from the Philippines^16^. QZNV is the only hantavirus reported hitherto in a frugivorous bat species (family Pteropodidae).

Several orthohantaviruses hosted by murid and cricetid rodents cause either hemorrhagic fever with renal syndrome (HFRS) in Europe and Asia^17–19^ or hantavirus cardiopulmonary syndrome (HCPS) in the Americas^20,21^. HFRS varies in clinical severity from mild to life threatening, with mortality ranging from <1% to ≥15%^22,23^, whereas HCPS is generally severe, and despite intensive care treatment, mortality rates are 25% or higher^24,25^. Humans are typically infected with rodent-borne orthohantaviruses by the respiratory route via inhalation of aerosolized excretions or secretions^26,27^. Transmission of hantaviruses from person-to-person has been reported only with Andes virus in Argentina^28^ and Chile^29,30^.

The pathogenicity of the newfound shrew-, mole- and bat-borne hantaviruses is unknown. That is, despite a report of IgG antibodies against recombinant nucleocapsid proteins of shrew-borne hantaviruses in humans from Côte d’Ivoire and Gabon^31^, there is no definitive proof that any of the recently reported orthohantaviruses, thottimviruses, loanviruses and mobatviruses, harbored by shrews, moles and bats, cause clinically identifiable diseases or syndromes in humans^2^.

This multi-institutional study represents an extended search for novel bat-borne loanviruses and mobatviruses to better understand their geographic distribution and host diversification. Our data indicate that a newly identified mobatvirus in the Stoliczka’s Asian trident bat (*Aselliscus stoliczkanus*) is genetically distinct and phylogenetically related to other bat-borne mobatviruses, suggesting that primordial hantaviruses may have been hosted by ancestral bats.

## Results

### Hantavirus detection

**Figure 1:**
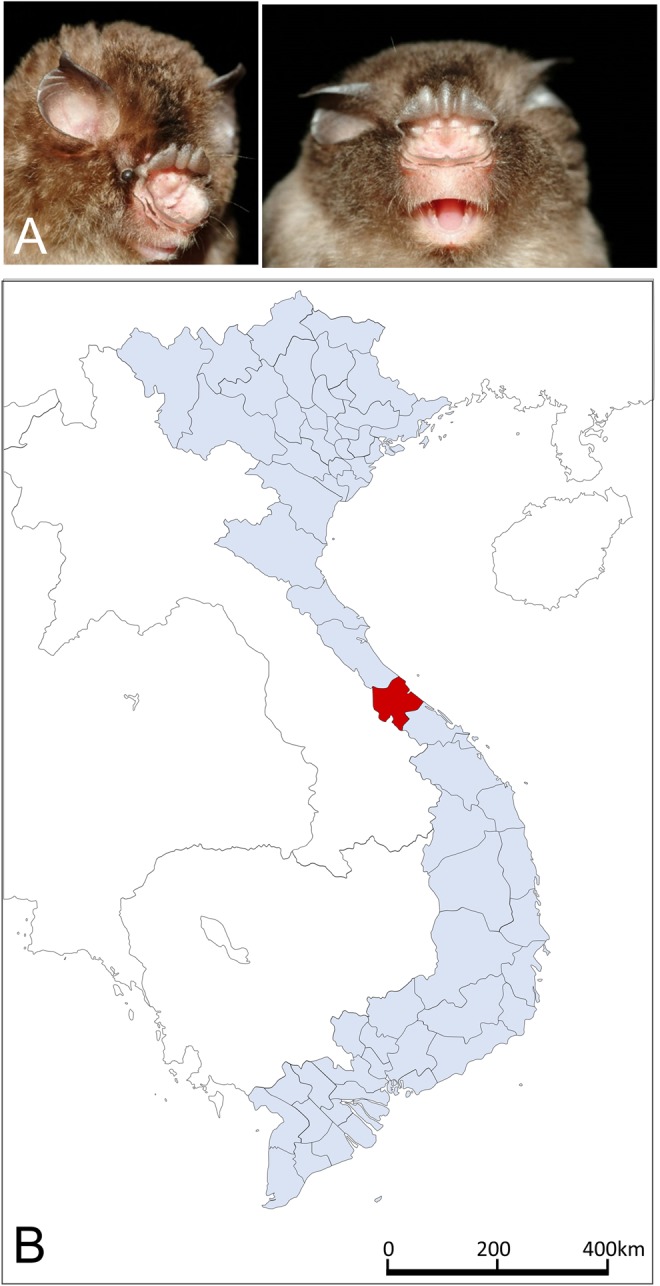
(**A**) Stoliczka’s Asian trident bat (*Aselliscus stoliczkanus*). (**B**) Map of Vietnam, showing Quảng Trị Province (colored red), where a mobatvirus-infected Stoliczka’s Asian trident bat was captured in Đakrông Nature Reserve.

Repeated attempts to detect hantavirus RNA were successful in only one of the 215 lung specimens (Supplemental Table 1), despite using PCR protocols that led to the discovery of other bat-borne hantaviruses. Sequence analysis of the full-length S-, M- and L-genomic segments showed a novel hantavirus, designated Đakrông virus (DKGV), in one of two Stoliczka’s Asian trident bats (Fig. 1A), captured in Đakrông Nature Reserve (16.6091N, 106.8778E) in Quảng Trị Province (Fig. 1B), in August 2013.

### Sequence analysis

The overall genomic organization of DKGV was similar to that of other hantaviruses. A nucleocapsid (N) protein of 427 amino acids was encoded by the 1,746-nucleotide S segment, beginning at position 58, and with a 405-nucleotide 3′-noncoding region (NCR). The hypothetical NSs open reading frame was not found.

A glycoprotein complex (GPC) of 1,127 amino acids was encoded by the 3,622-nucleotide M segment, starting at position 21, and with a 218-nucleotide 3′-NCR. Two potential N-linked glycosylation sites were found in the Gn at amino acid positions 344 and 396 and one in the Gc at position 924. Another possible site was present at amino acid position 133 of the Gn. For comparison, the amino acid positions of N-linked glycosylation sites of other representative hantaviruses are summarized in Table 1. Also, the highly conserved WAASA amino-acid motif was found at amino acid positions 641–645. The full-length Gn/Gc amino acid sequence similarity was highest between DKGV and LAIV (73.0%) (Table 2).

**Table 1:** Potential N-linked glycosylation sites in the Gn and Gc glycoproteins of DKGV strain VN2913B72 and representative bat-, rodent-, shrew- and mole-borne hantaviruses.

Reservoir host	Virus and strain	Gn						Gc
Bat	DKGV VN2913B72		133?		344	396		924
	XSV VN1982B4		143	230	345	397	540	925
	LAIV BT20		133		344	396	539, 606	924
	LQUV Ra-25		134		349?	441	544, 569, 608?	883, 929
	BRNV 7/2012/CZE	96	137?		313, 352?	444		931, 1053
	QZNV MT1720/1657		133		300	399	564	928
Murid rodent	DOBV/BGDV Greece		134	235	347	399	518, 562	928
	HTNV 76-118		134	235	347	399	609	928
	SANGV SA14		134	235	347	399	518, 562, 566	928
	SEOV HR80-39		132	233	345	397	560	926
	SOOV SOO-1		134	235	347	399	609	928
Cricetid rodent	PUUV Sotkamo		142		357	409		898, 937
	MUJV 11-1		136		351	403		892, 931
	PHV PH-1		139		353	405	527	933
	TULV M5302v		140		355?	407	583	935
	SNV NMH10		138		351	403		931
	ANDV Chile9717869		138		350	402	524	930
Crocidurine shrew	BOWV VN1512	23	142		355	407	526, 701	936, 1060?
	JJUV 10-11		142		355	407		936, 1058
	MJNV Cl05-11		133	288	387			915, 1078
	TPMV VRC66412		134	289	388		505, 585	916
Soricine shrew	ASIV Drahany		138		351	403	566	932
	ARTV MukawaAH301		138		351	403		932
	CBNV CBN-3		138		351	403		932
	YKSV Si-210		138		351	403	566	932
Mole	RKPV MSB57412		135		349	401	577	890, 929
	OXBV Ng1453		138		353	405	524, 617, 623	934
	ASAV N10		138		352	404		933
	NVAV Te34	101	133		344	396	539	924

Đakrông virus (DKGV) VN2913B72, Xuân Sơn virus (XSV) VN1982B4, Láibīn virus (LAIV) BT20, Lóngquán virus (LQUV) Ra-25, Brno virus (BRNV) 7/2012/CZE and Quezon virus (QZNV) MT1720/1657 were detected in bats, Dobrava-Belgrade virus (DOBV/BGDV) Greece, Hantaan virus (HTNV) 76-118, Sangassou virus (SANGV) SA14, Seoul virus (SEOV) HR80-39 and Soochong virus (SOOV) SOO-1 in murid rodents, Puumala virus (PUUV) Sotkamo, Muju virus (MUJV) 11-1, Prospect Hill virus (PHV) PH-1, Tula virus (TULV) M5302v, Sin Nombre virus (SNV) NMH10 and Andes virus (ANDV) Chile9717869 in cricetid rodents, Bowé virus (BOWV) VN1512, Jeju virus (JJUV) 10-11, Imjin virus (MJNV) Cl05-11 and Thottapalayam virus (TPMV) VRC66412 in crocidurine shrews, Asikkala virus (ASIV) Drahany, Artybash virus (ARTV) MukawaAH301, Cao Bằng virus (CBNV) CBN-3 and Yákèshí virus (YKSV) Si-210 in soricine shrews and Rockport virus (RKPV) MSB57412, Oxbow virus (OXBV) Ng1453, Asama virus (ASAV) N10 and Nova virus (NVAV) Te34 in moles.

**Table 2:** Nucleotide (nt) and amino acid (aa) sequence similarities of the coding regions of the full-length S, M and L segments of DKGV strain VN2913B72 and representative bat-, rodent-, shrew- and mole-borne hantaviruses.

Reservoir host	Virus and strain	S segment		M segment		L segment	
		1284 nt	427 aa	3384 nt	1127 aa	6438 nt	2145 aa
Bat	XSV VN1982B4	69.9%	76.5%	66.9%	69.3%	71.3%	79.4%
	LAIV BT20	70.3%	76.8%	69.2%	73.0%	73.4%	81.2%
	LQUV Ra-25	62.0%	59.6%	55.4%	45.9%	69.8%	72.2%
	HUPV Pa-1	64.0%	63.1%	—	—	68.8%	79.0%
	BRNV 7/2012/CZE	59.7%	56.7%	54.6%	45.6%	65.6%	66.1%
	QZNV MT1720/1657	55.9%	64.6%	58.4%	53.3%	66.3%	69.4%
Murid rodent	DOBV/BGDV Greece	57.2%	53.2%	53.7%	45.9%	64.6%	65.4%
	HTNV 76-118	57.0%	52.5%	53.7%	43.6%	64.6%	65.3%
	SANGV SA14	58.0%	52.2%	53.8%	45.6%	64.3%	65.0%
	SEOV HR80-39	56.3%	51.1%	53.7%	43.4%	64.9%	65.4%
	SOOV SOO-1	57.5%	53.9%	53.5%	43.9%	65.0%	65.0%
Cricetid rodent	PUUV Sotkamo	58.0%	53.2%	54.2%	46.0%	64.8%	65.1%
	TULV M5302v	59.1%	51.8%	54.8%	46.4%	64.0%	64.8%
	PHV PH-1	59.1%	53.2%	54.9%	47.2%	62.9%	65.1%
	SNV NMH10	56.9%	50.4%	54.7%	48.2%	63.2%	64.7%
	ANDV Chile9717869	57.1%	52.7%	54.6%	47.0%	63.9%	64.9%
Soricine shrew	CBNV CBN-3	57.4%	53.0%	54.5%	44.9%	64.3%	65.6%
	ARRV MSB734418	53.3%	44.0%	—	—	65.4%	65.2%
	JMSV MSB144475	55.3%	50.2%	58.1%	50.6%	64.2%	65.9%
	ARTV MukawaAH301	56.2%	50.9%	54.1%	44.8%	63.7%	64.8%
	SWSV mp70	55.8%	51.4%	60.5%	56.6%	62.5%	62.4%
	KKMV MSB148794	56.0%	51.2%	53.5%	44.0%	63.9%	64.9%
	QHSV YN05 284 S	56.7%	50.5%	56.6%	48.3%	71.4%	75.2%
	YKSV Si-210	56.4%	50.0%	53.9%	44.9%	63.2%	65.1%
	TGNV Tan826	55.1%	47.6%	—	—	67.5%	67.2%
	AZGV KBM15	57.9%	54.6%	55.1%	44.5%	63.5%	65.2%
Crocidurine shrew	JJUV SH42	56.4%	49.8%	54.1%	45.1%	63.7%	63.7%
	BOWV VN1512	56.7%	49.9%	55.1%	43.6%	62.3%	63.8%
	MJNV Cl05-11	53.6%	45.8%	51.2%	40.0%	63.3%	64.9%
	TPMV VRC66412	54.4%	44.9%	51.4%	42.1%	63.6%	64.8%
Myosoricine shrew	ULUV FMNH158302	55.7%	49.1%	54.2%	42.6%	63.1%	64.5%
	KMJV FMNH174124	55.4%	48.7%	54.9%	45.4%	63.3%	64.1%
Mole	RKPV MSB57412	57.7%	53.2%	55.7%	46.0%	63.7%	64.4%
	OXBV Ng1453	56.0%	51.1%	53.6%	44.2%	63.1%	64.4%
	ASAV N10	57.4%	51.3%	53.8%	45.3%	64.1%	64.6%
	NVAV Te34	60.7%	57.0%	60.7%	56.4%	65.5%	67.0%

Huángpí virus (HUPV) was detected in a bat species, Ash River virus (ARRV), Azagny virus (AZGV), Jemez Springs virus (JMSV), Kenkeme virus (KKMV), Qian Hu Shan virus (QHSV), Seewis virus (SWSV) and Tanganya virus (TGNV) were detected in soricine shrews, and Kilimanjaro virus (KMJV) and Uluguru virus (ULUV) were detected in myosoricine shrews. The other abbreviations of virus names are the same as in Table 1. Three bat-borne hantaviruses from Africa, Makokou virus (MAKV), Magboi virus (MGBV) and Mouyassué virus (MOYV), are not included because only relatively short regions of the L segment were available for analysis. Hyphens indicate no available sequence data.

Analysis of the 6,535-nucleotide L segment, which encoded a 2,145-amino acid RNA-dependent RNA polymerase (RdRP), showed the highly conserved A, B, C, D and E motifs, The functional constraints on the RdRP were evidenced by the overall high nucleotide and amino acid sequence similarity of 60% or more in the L segment between DKGV and other hantaviruses (Table 2).

Comparison of the full-length S, M and L segments indicated amino acid sequence similarities ranging from 45.6% (BRNV GPC) to 81.2% (LAIV RdRP) between DKGV and representative bat-borne hantaviruses (Table 2), and showed that DKGV differed at the amino acid level by 30% or more from nearly all rodent- and shrew-borne orthohantaviruses.

### Nucleocapsid and glycoprotein secondary structures

**Figure 2:**
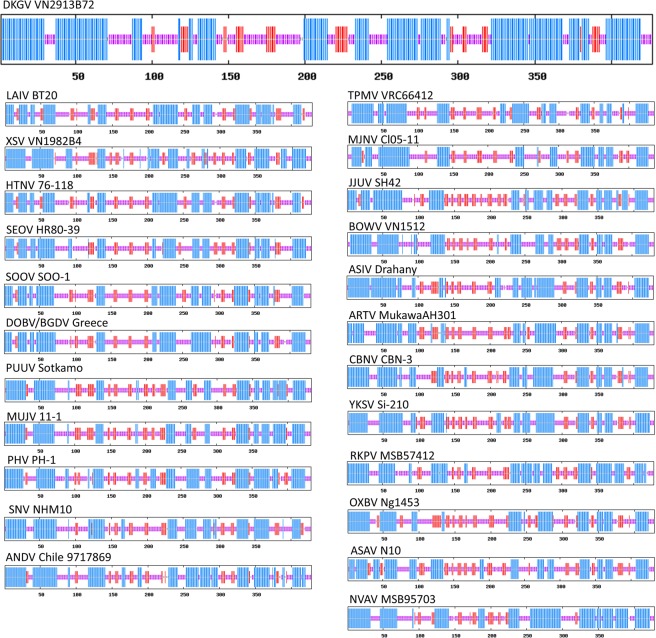
Comparison of consensus secondary structures of entire nucleocapsid (N) proteins of DKGV VN2913B72 (large top panel) and other representative hantaviruses (smaller panels), predicted using several methods at the NPS@ structure server^14^. N protein structures are shown for bat-borne mobatviruses (DKGV VN2913B72, LAIV BT20 and XSV VN1982B4), rodent borne orthohantaviruses (HTNV 76-118, SEOV HR80-39, SOOV SOO-1, DOBV/BGDV Greece, PUUV Sotkamo, MUJV 11-1, PHV PH-1, SNV NMH10 and ANDV Chile9719869) and shrew-borne thottimviruses (TPMV VRC66412 and MJNV Cl05-11), shrew-borne orthohantaviruses (JJUV SH42, BOWV VN1512, ASIV Drahany, ARTV MukawaAH301, CBNV CBN-3 and YKSV Si-210), and mole-borne orthohantaviruses (RKPV MSB57412, OXBV Ng1453 and ASAV N10) and mole-borne mobatvirus (NVAV MSB95703). Blue bars represent α-helices, red bars β-strands, and purple indicate random coil and unclassified structures, respectively. Abbreviations: ANDV, Andes virus; ARTV, Artybash virus; ASAV, Asama virus; ASIV, Asikkala virus; BOWV, Bowé virus; CBNV, Cao Bằng virus; DKGV, Đakrông virus; DOBV/BGDV, Dobrava-Belgrade virus; HTNV, Hantaan virus; JJUV, Jeju virus; LAIV, Láibīn virus; MJNV, Imjin virus; MUJV, Muju virus; NVAV, Nova virus; OXBV, Oxbow virus; PHV, Prospect Hill virus; PUUV, Puumala virus; RKPV, Rockport virus; SEOV, Seoul virus; SNV, Sin Nombre virus; SOOV, Soochong virus; TPMV, Thottapalayam virus; XSV, Xuân Sơn virus; YKSV, Yákèshí virus.

**Figure 3:**
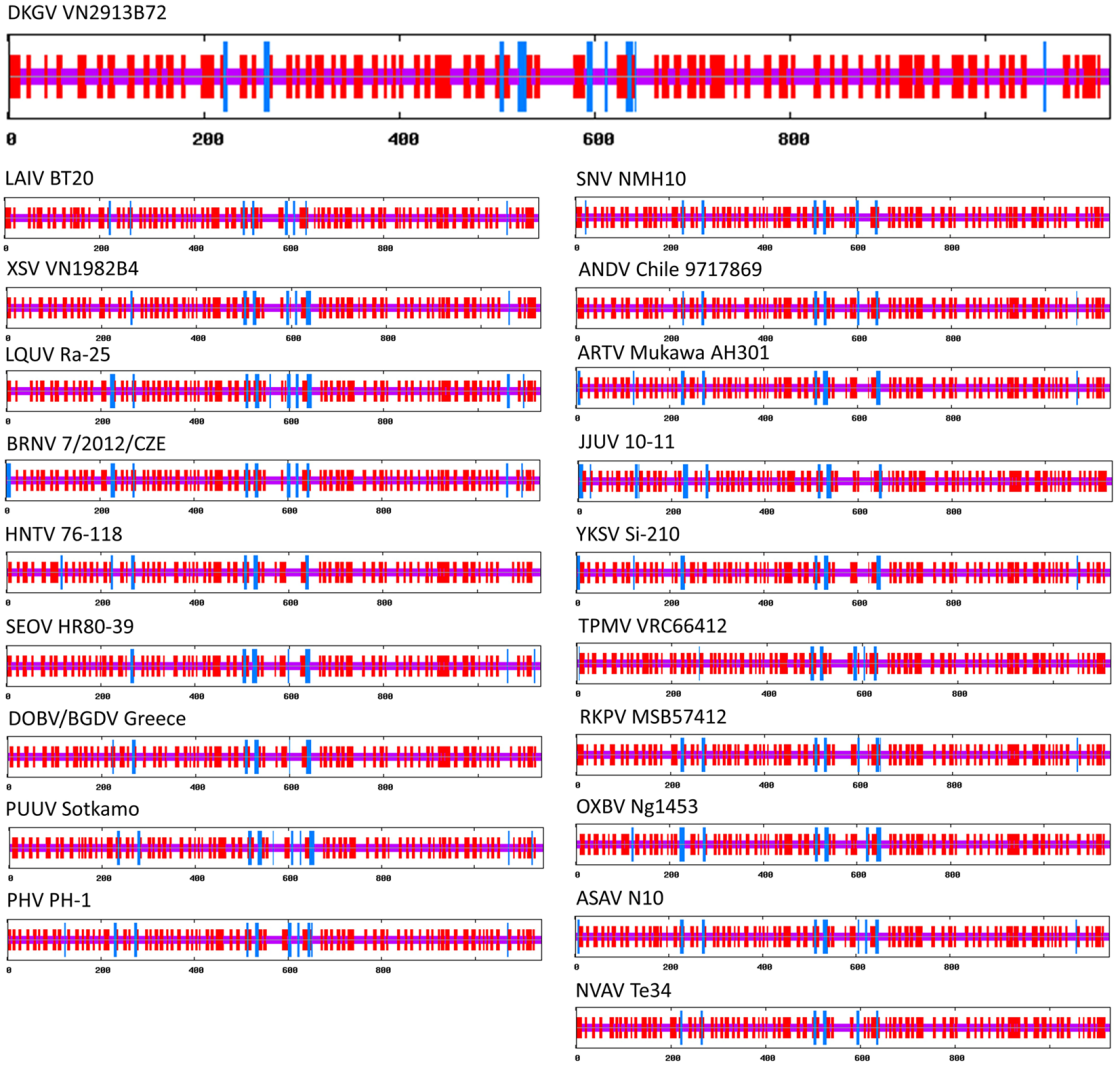
Comparison of consensus secondary structures of entire envelope glycoproteins (G) of DKGV VN2913B72 (large top panel) and other representative hantaviruses (smaller panels), predicted at the NPS@ structure server^44^. G protein structures are shown for bat-borne mobatviruses (DKGV VN2913B72, LAIV BT20 and XSV VN1982B4), bat-borne loanviruses (LQUV Ra-25 and BRNV 7/2012/CZE), rodent borne orthohantaviruses (HTNV 76-118, SEOV HR80-39, DOBV/BGDV Greece, PUUV Sotkamo, PHV PH-1, SNV NMH10 and ANDV Chile 9717869), shrew-borne orthohantaviruses (ARTV MukawaAH301, JJUV 10-11 and YKSV Si-210), shrew-borne thottimvirus (TPMV VRC66412), mole-borne orthohantaviruses (RKPV MSB57412, OXBV Ng1453, ASAV N10) and mole-borne mobatvirus (NVAV Te34). Blue bars represent α-helices, red bars β-strands, and purple indicate random coil and unclassified structures, respectively. Abbreviations: BRNV, Brno virus; LQUV, Lóngquán virus; other abbreviations of virus names, as in Fig. 2 legend.

Secondary structure analysis of the N and Gn/Gc proteins indicated similarities and differences between DKGV and representative rodent-, shrew-, mole- and bat-borne hantaviruses (Figs 2 and 3).

The DKGV N protein secondary structure, comprising 53.16% α-helices, 10.07% β-sheets and 35.13% random coils, resembled that of other hantavirus N proteins. A two-domain, primarily α-helical structure joined by a central β-pleated sheet, was observed. Although the N-terminal domain length was nearly the same, structural changes were evident in the central β-pleated sheet and adjoining C-terminal α-helical domain, according to the phylogenetic relationships of the N proteins.

That is, the N protein comprised two α-helical domains and a central β-pleated sheet (Fig. 2) irrespective of the low amino acid sequence similarity among the orthohantaviruses and thottimviruses. However, the central β-pleated sheet motif and RNA-binding region (amino acid positions 175 to 217) of DKGV differed from that of other hantaviruses, which resembled that of murid rodent-borne orthohantaviruses (Fig. 2).

**Figure 4:**
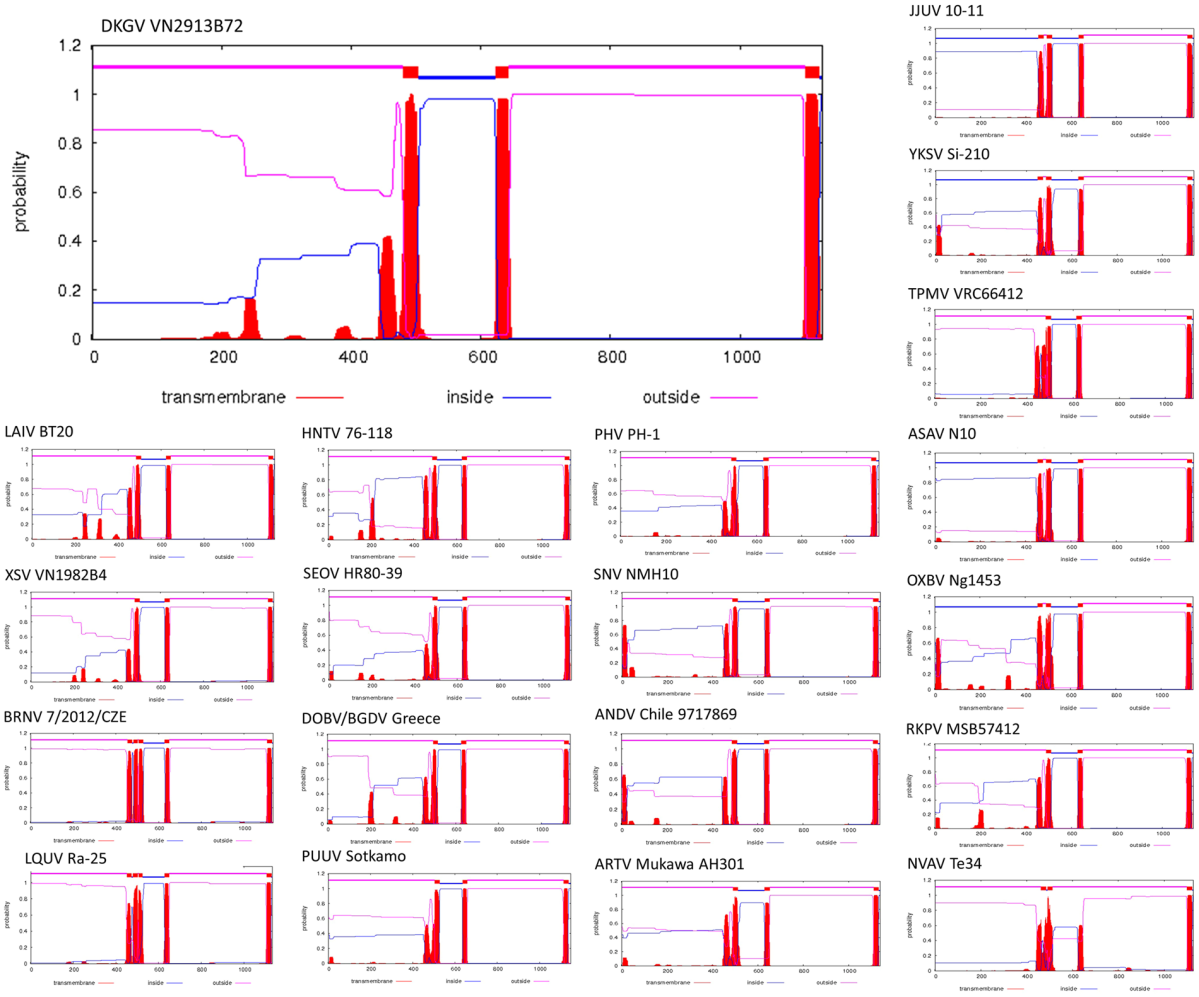
Comparison of consensus predicted transmembrane helices of the envelope glycoprotein complex (GPC) of DKGV VN2913B72 and other representative hantaviruses. GPC structures are shown for mobatviruses hosted by bats (DKGV VN2913B72, LAIV BT20 and XSV VN1982B4) and mole (NVAV Te34), bat-borne loanviruses (LQUV Ra-25 and BRNV 7/2012/CZE), orthohantaviruses harbored by rodents (HTNV 76-118, SEOV HR80-39, DOB/BGDV Greece, PUUV Sotkamo, PHV PH-1, SNV NMH10 and ANDV Chile 9717869), shrews (JJUV 10-11, ARTV Mukawa AH301 and YKSV Si-210) and moles (RKPV MSB57412, OXBV Ng1453, ASAV N10) and shrew-borne thottimvirus (TPMV VRC66412). Red bars represent transmembrane structure, and blue and pink lines indicate inside and outside membrane, respectively.

The DKGV glycoprotein secondary structure, comprising 3.82% α-helices, 40.20% β-sheets and 55.99% random coils, resembled that of other hantavirus glycoproteins (Fig. 3). Also, the four transmembrane helices of the DKGV glycoprotein resembled that of other hantaviruses (Fig. 4), and the putative fusion loop (WGCNPVD) and zinc finger domain (**C**VV**C**TRECSCTEELKA**H**NEH**C**IQGS**C**PY **C**MRDLHPSQHVLTE**H**YKT**C)** were observed at residues 760–766 and 542–588, respectively.

### Hantavirus phylogeny

**Figure 5:**
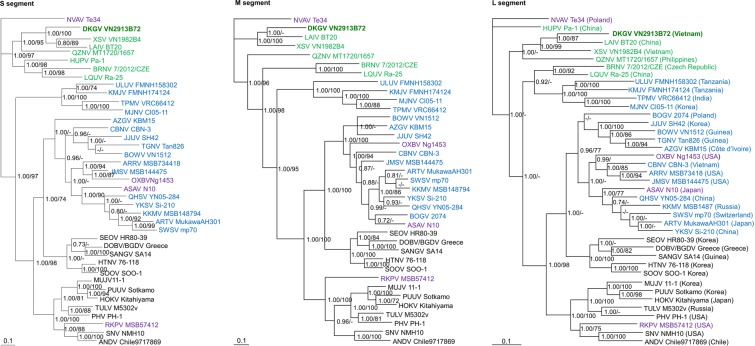
Phylogenetic trees, based on 1,284-, 3,384- and 6,438-nucleotide regions of the S-, M- and L-genomic segments, respectively, of Đakrông virus (DKGV VN2913B72) (S: MG663534, M: MG663535 and L: MG663536) from Stoliczka’s Asian trident bat, generated by the Bayesian Markov chain Monte Carlo estimation method, under the GTR + I + Γ model of evolution. The phylogenetic position of DKGV is shown in relation to other chiropteran- and mole-borne mobatviruses, including Láibīn virus (LAIV BT20, S: KM102247; M: KM102248; L: KM102249) from *Taphozous melanopogon*, Xuân Sơn virus (XSV VN1982B4, S: KC688335; M: KU976427; L: JX912953) from *Hipposideros pomona*, Quezon virus (QZNV MT1720/1657, S: KU950713; M: KU950714; L: KU950715) from *Rousettus amplexicaudatus*, and Nova virus (NVAV Te34, S: KR072621; M: KR072622; L: KR072623) from *Talpa europaea*. Loanviruses including Brno virus (BRNV 7/2012/CZE, S: KX845678; M: KX845679; L: KX845680) from *Nyctalus noctula*, Lóngquán viruse (LQUV Ra-25, S: JX465415; M: JX465397; L: JX465381) from *Rhinolophus affinis*, and Huángpí viruse (HUPV Pa-1, S: JX473273; L: JX465369) from *Pipistrellus abramus* were shown. Also shown are shrew-borne orthohantaviruses including Ash River virus (ARRV MSB734418, S: EF650086; L: EF619961) from *Sorex cinereus*, Artybash virus (ARTV MukawaAH301, S: KF974360; M: KF974359; L: KF974361) from *Sorex caecutiens*, Azagny virus (AZGV KBM15, S: JF276226; M: JF276227; L: JF276228) from *Crocidura obscurior*, Boginia virus (BOGV 2074, M: JX990966; L: JX990965), Bowé virus (BOWV VN1512, S: KC631782; M: KC631783; L: KC631784) from *Crocidura douceti*, Cao Bằng virus (CBNV CBN-3, S: EF543524; M: EF543526; L: EF543525) from *Anourosorex squamipes*, Jeju virus (JJUV SH42, S: HQ663933; M: HQ663934; L: HQ663935) from *Crocidura shantungensis*, Jemez Springs virus (JMSV MSB144475, S: FJ593499; M: FJ593500; L: FJ593501) from *Sorex monticolus*, Kenkeme virus (KKMV MSB148794, S: GQ306148; M: GQ306149; L: GQ306150) from *Sorex roboratus*, Qian Hu Shan virus (QHSV YN05-284, S: GU566023; M: GU566022; L: GU566021) from *Sorex cylindricauda*, Seewis virus (SWSV mp70, S: EF636024; M: EF636025; L: EF636026) from *Sorex araneus*, Tanganya virus (TGNV Tan826, S: EF050455; L: EF050454) from *Crocidura theresea* and Yákèshí virus (YKSV Si-210, S: JX465423; M: JX465403; L: JX465389) from *Sorex isodon*, as well as mole-borne orthohantaviruses including Asama virus (ASAV N10, S: EU929072; M: EU929075; L: EU929078) from *Urotrichus talpoides*, Oxbow virus (OXBV Ng1453, S: FJ5339166; M: FJ539167; L: FJ593497) from *Neurotrichus gibbsii*, and Rockport virus (RKPV MSB57412, S: HM015223; M: HM015222; L: HM015221) from *Scalopus aquaticus*. Shrew-borne thottimviruses include Thottapalayam virus (TPMV VRC66412, S: AY526097; L: EU001330) from *Suncus murinus*, Imjin virus (MJNV Cl05-11, S: EF641804; M: EF641798; L: EF641806) from *Crocidura lasiura*, Uluguru virus (ULUV FMNH158302, S: JX193695; M: JX193696; L: JX193697) from *Myosorex geata*, and Kilimanjaro virus (KMJV FMNH174124, S: JX193698; M: JX193699; L: JX193700) from *Myosorex zinki*. Other taxa include rodent borne orthohantaviruses, Andes virus (ANDV Chile9717869, S: AF291702; M: AF291703; L: AF291704), Sin Nombre virus (SNV NMH10, S: NC_005216; M: NC_005215; L: NC_005217), Dobrava-Belgrade virus (DOBV/BGDV Greece, S: NC_005233; M: NC_005234; L: NC_005235), Hantaan virus (HTNV 76-118, S: NC_005218; M: NC_005219; L: NC_005222), Hokkaido virus (HOKV Kitahiyama, S: AB675463; M: AB676848; L: AB712372), Muju virus (MUJV 11-1, S: JX028273; M: JX028272; L:JX028271), Prospect Hill virus (PHV PH-1, S: Z49098; M: X55129; L: EF646763), Puumala virus (PUUV Sotkamo, S: NC_005224; M: NC_005223; L: NC_005225), Sangassou virus (SANGV SA14, S: JQ082300; M: JQ082301; L: JQ082302), Seoul virus (SEOV HR80-39, S: NC_005236; M: NC_005237; L: NC_005238), Soochong virus (SOOV SOO-1, S: AY675349; M: AY675353; L: DQ056292), and Tula virus (TULV M5302v, S: NC_005227; M: NC_005228; L: NC_005226). The numbers at each node are Bayesian posterior probabilities (>0.7, left of slash) based on 150,000 trees: two replicate Markov chain Monte Carlo runs, consisting of six chains of 10 million generations each sampled every 100 generations with a burn-in of 25,000 (25%) and bootstrap value (>70%, right of slash) based on 1000 bootstrap replicates. Scale bars indicate nucleotide substitutions per site. Bat-borne hantaviruses are shown in green lettering (DKGV VN2913B72 shown in bold), shrew-borne hantaviruses in blue, mole-borne hantaviruses in purple and rodent borne hantaviruses in black.

DKGV was distinct from other hantaviruses in phylogenetic trees, based on S-, M- and L-segment sequences using the Markov chain Monte Carlo (MCMC) Bayesian methods (Fig. 5). In all analyses, DKGV and LAIV shared a common ancestry. The basal position of mobatviruses and loanviruses in phylogenetic trees suggested that primordial hantaviruses may have been hosted by ancestral bats (Fig. 5).

### Host phylogeny

**Figure 6:**
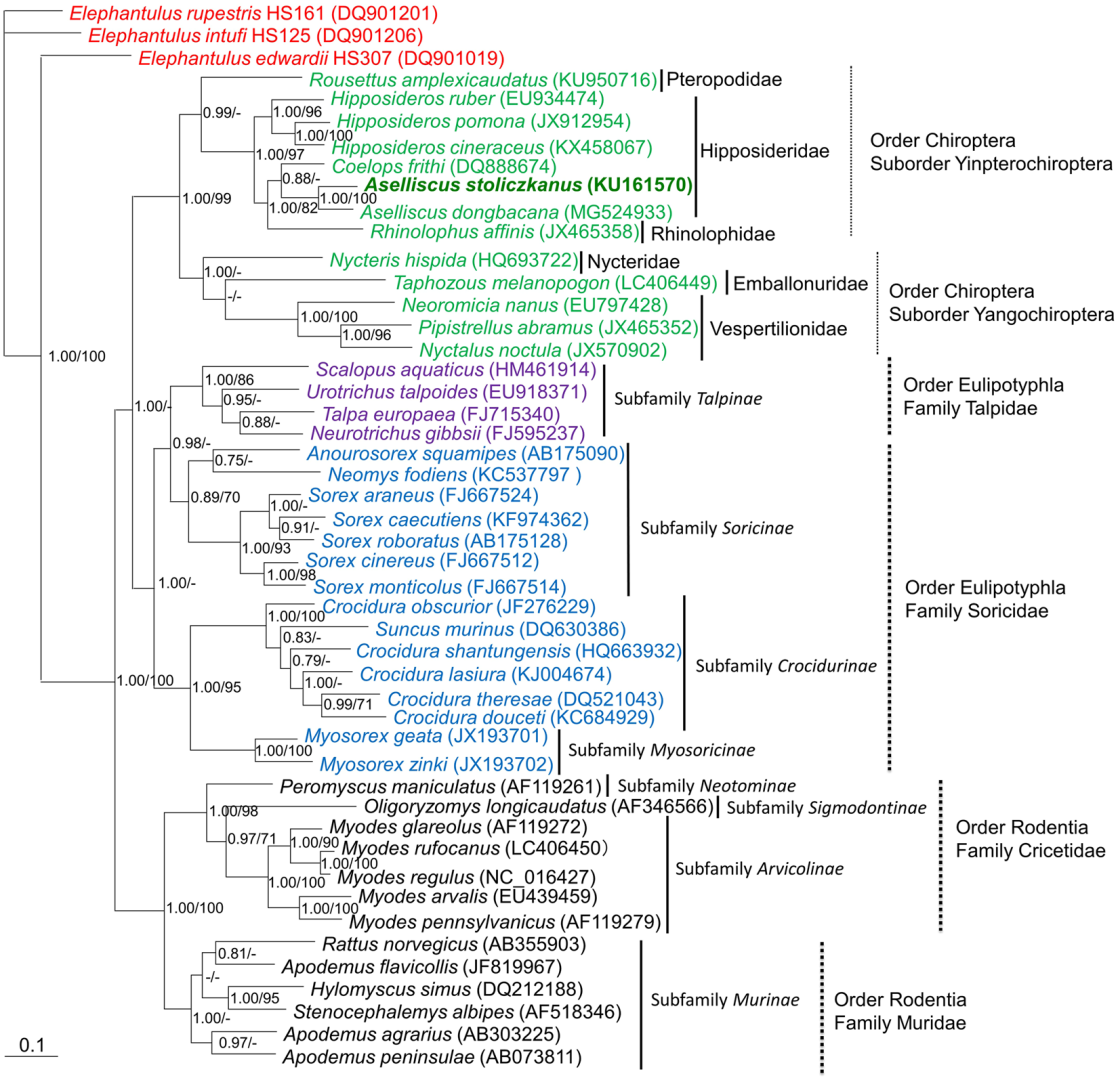
Bayesian phylogenetic tree, based on the 1,140-nucleotide cytochrome *b* (Cyt *b*) region of mtDNA of small mammals within the order Eulipotyphla (families Talpidae and Soricidae), order Rodentia (families Muridae and Cricetidae) and order Chiroptera, suborder Yinpterochiroptera (families Pteropodidae, Hipposideridae, Rhinolophidae) and suborder Yangochiroptera (families Nycteridae, Emballonuridae and Vespertilionidae). The tree was rooted using *Elephantulus* (order Macroscelidea, GenBank accession numbers DQ901019, DQ901206 and DQ901201). Numbers at nodes indicate posterior probability values (>0.7, left of slash) based on 150,000 trees: two replicate Markov chain Monte Carlo runs, consisting of six chains of 10 million generations each sampled every 100 generations with a burn-in of 25,000 (25%) and bootstrap value (>70%, right of slash) based on 1000 bootstrap replicates. Scale bars indicate nucleotide substitutions per site. Letterings for taxa are shown in green for bats, blue for shrews, purple for moles, black for rodents and red for *Elephantulus*. The GenBank accession number for the Cyt *b* sequence for *Aselliscus stoliczkanus* is KU161570.

Taxonomic classification of the bats was confirmed by PCR amplification and sequencing of the cytochrome *b* (Cyt *b*) and cytochrome c oxidase I (COI) genes. Morphologically indistinguishable *Aselliscus stoliczkanus* and *Aselliscus dongbacana* were identified by Cyt *b* (KU161558–KU161575 and MG524933–MG524935) and COI (LC406430–LC406448) gene sequence analysis. Phylogenetic analysis of bats belonging to the suborders Yinpterochiroptera and Yangochiroptera resembled that of DKGV and other bat-borne hantaviruses (Fig. 6).

### Co-phylogeny of hantavirus and host

**Figure 7:**
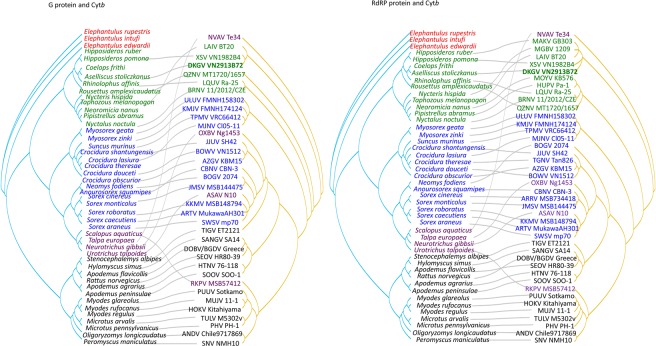
Tanglegram comparing the phylogenies of hantaviruses and their chiroptera, eulipotyphla, and rodent hosts. The host tree on the left was based on cytochrome *b* (Cyt *b*) gene sequences, while the hantavirus tree on the right was based on the amino acid sequences of glycoprotein (**A**) and RNA-dependent RNA polymerase (**B**), respectively. Letterings for taxa are shown in green for bats, blue for shrews, purple for moles, black for rodents and red for *Elephantulus* in the each left panel. Bat-borne hantaviruses are shown in green lettering (DKGV VN2913B72 shown in bold), shrew-borne hantaviruses in blue, mole-borne hantaviruses in purple and rodent borne hantaviruses in black in the each right panel. The host species and viruses relationship (Cyt *b* and each segment sequence accession number) were listed in Supplemental Table 3.

Segregation of hantaviruses according to the subfamily of their reservoir hosts was demonstrated by co-phylogeny mapping, using consensus trees based on the Gn/Gc glycoprotein and RdRP protein amino acid sequences (Fig. 7). The phylogenetic positions of DKGV and other mobatviruses (XSV and MAKV) mirrored the phylogenetic relationships of their Hipposideridae hosts. By contrast, the phylogenetic positions of LAIV from *Taphozous melanopogon*, QZNV from *Rousettus amplexicaudatus* and LQUV from *Rhinolophus affinis* were mismatched between virus and host species tanglegram.

## Discussion

The Stoliczka’s Asian trident bat, one of three species in the genus *Aselliscus*, is found throughout Southeast Asia. The Dong Bac’s trident bat (*Aselliscus dongbacana*), a closely related species, overlaps in body size, distribution, echolocation and habitat^32^. However, we failed to detect hantavirus RNA in this latter species. As in previous studies, in which only one or two individual bats were found to be infected^7,12,13,16^, hantavirus RNA was detected in a single Stoliczka’s Asian trident bat. Technical issues (such as primer mismatches, suboptimal cycling conditions, limited tissues and degraded RNA), as well as the restricted transmission and/or immune-mediated clearance of hantavirus infection in bats, are possible contributing factors.

By virtue of the N protein and Gc glycoprotein sequence divergence between DKGV and other hantaviruses, as well as the unique host species, DKGV likely represents a new hantavirus species. A three-dimensional, bi-lobed protein architecture for RNA binding was suggested by the DKGV N protein secondary structure and that of other bat-borne hantaviruses. The fusion loop and zinc finger domain of the glycoprotein suggested that DKGV and other bat-borne loanvirus and mobatviruses have similar mechanisms of protein modification with rodent-borne orthohantaviruses^33,34^. On the other hand, the envelope GPC is implicated in virus attachment and cell entry. Further insights for receptor and receptor binding sites of hantaviruses harbored by shrews, moles and bats will contribute to a deeper understanding about their host specificities.

Recently, far greater genetic diversity than those in rodents have been detected in orthohantaviruses harbored by shrews and moles of multiple species, belonging to five subfamilies (Soricinae, Crocidurinae, Myosoricinae, Talpinae and Scalopinae) within the order Eulipotyphla, in Europe, Asia, Africa and/or North America. Similarly, hantavirus RNA has been detected in tissues of several bat species belonging to the families Emballonuridae, Nycteridae and Vespertilionidae (suborder Yangochiroptera) and the families Rhinolophidae, Hipposideridae and Pteropodidae (suborder Yinpterochiroptera). It is unclear to what extent spillover or host switching is responsible for the overall low prevalence of hantavirus infection in only 14 bat species to date, and the inability to detect hantavirus RNA in nearly 100 other bat species analyzed to date^7–16^.

Previously, the overall congruence between gene phylogenies of rodent-borne orthohantaviruses and their hosts led to the conjecture that orthohantaviruses had co-evolved with their reservoir hosts^4^. However, recent studies, based on co-phylogenetic reconciliation and estimation of evolutionary rates and divergence times, conclude that local host-specific adaptation and preferential host switching account for the phylogenetic similarities between hantaviruses and their mammalian hosts^35,36^. Although our co-phylogenetic analysis also indicates that bat-borne loanvirus and mobatviruses and their host species have not co-diverged, the availability of whole genome sequences for only four of the 10 bat-borne hantaviruses presented a significant limitation. Thus, future efforts must focus on obtaining full-length genomes of newfound hantaviruses, particularly those harbored by bats in southeast Asia and Africa, to gain additional insights into the phylogeography and evolutionary origins of viruses in the family *Hantaviridae*.

## Materials and Methods

### Ethics statement

Field procedures and protocols for trapping, euthanasia and tissue processing, conforming to the guidelines of the American Society of Mammalogists^37,38^, were approved by the Ministry of Agriculture and Rural Development in Vietnam. Moreover, permission for this study was obtained from the Vietnam Administration of Forest, belonging to the Ministry of Agriculture and Rural Development, before collecting bat specimens (permission numbers: 1492/TCLN-BTTN; 701/TCLN-BTTN; 389/TCLN-BTTN; 767/TCLN-BTTN). Also, the Institutional Animal Care and Use Committee, of the National Institute of Infectious Diseases, reviewed and approved the field protocols and experimental procedures (permission number: 112152).

### Trapping

Mist nets and harp traps were used to trap bats, during December 2012 to June 2014, in Xuân Lien Nature Reserve (19.8736N, 105.2156E) in Thanh Hóa Province; Vĩnh Cửu Nature Reserve, recently renamed Đồng Nai Culture and Nature Reserve (11.3808N, 107.0622E), in Đồng Nai Province; Khau Ca Nature Reserve (22.8381N, 105.1161E) in Hà Giang Province; Bắc Hướng Hóa Nature Reserve and Đakrông Nature Reserve in Quảng Trị Province; Nà Hang Nature Reserve (22.3532N, 105.4197E) in Tuyên Quang Province; and near Hoàng Liên National Park (22.2833N, 103.9219E) in Lào Cai Province. Live-caught bats were euthanized and lung tissues were preserved in RNAlater® (Qiagen) until testing by RT-PCR.

### RNA extraction

Using the MagDEA RNA 100 Kit (Precision System Science, Matsudo, Japan)^39^, total RNA was extracted from lung tissues of 215 bats, representing 15 genera and 46 species in five families (Hipposideridae, Megadermatidae, Pteropodidae, Rhinolophidae and Vespertilionidae) (Supplemental Table 1). RNA was reverse transcribed to cDNA was synthesized using the PrimeScript II 1st strand cDNA Synthesis Kit (Takara bio, Otsu, Japan) and an oligonucleotide primer (OSM55F, 5′–TAGTAGTAGACTCC–3′) designed from conserved 5′–end of the S, M and L segments of hantaviruses^39^.

### RT-PCR and sequencing

Oligonucleotide primers previously used to detect hantaviruses^12,13,16,39–43^ were employed to amplify S, M and L segments (Supplemental Table 2). First-round PCR was performed in 20-μL reaction mixtures, containing 250 μM dNTP, 2.5 mM MgCl_2_, 1 U of Takara LA Taq polymerase Host Start version (Takara Bio) and 0.25 μM of each primer^16^. Second-round PCR was performed in 20-μL reaction mixtures, containing 200 μM dNTP, 2.5 mM MgCl_2_, 1 U of AmpliTaq 360 Gold polymerase (Life Technologies, Foster City, CA, USA) and 0.25 μM of each primer^16^. Initial denaturation at 95 °C for 1 min for first PCR or at 95 °C for 10 min for second PCR were followed by two cycles each of denaturation at 95 °C for 20 s, two-degree step-down annealing from 46 °C to 38 °C for 40 s, and elongation at 68 °C for 1 min, then 30 cycles of denaturation at 94 °C for 20 s, annealing at 42 °C for 40 s, and elongation at 68 °C for 1 min, in a Veriti thermal cycler (Life Technologies)^7,12,13,16,39^. PCR products, treated with ExoSAP-IT (Affymetrix, Santa Clara, CA, USA) according to the manufacturer’s instruction, were sequenced directly using an ABI 3730xl DNA Analyzer (Life Technologies)^16^.

### Secondary structure analysis

Full-length amino acid sequences were submitted to NPS@ structure server^44^ to predict secondary structures of the N protein and Gn/Gc glycoproteins^36^. Glycosylation and transmembrane sites were predicted at the NetNlyc 1.0 and Predictprotein^45^ and TMHMM version 2.0^46^, respectively. The program COILS^47^ was used to scan the N protein for expected coiled-coil regions^36^.

### Phylogenetic analysis

Maximum likelihood and Bayesian methods, implemented in RAxML Blackbox webserver^48^ and MrBayes 3.1^49^, under the best-fit GTR + I + Γ model of evolution and jModelTest version 2.1.6^50^, were used to generate phylogenetic trees^13^. Two replicate Bayesian Metropolis–Hastings MCMC runs, each consisting of six chains of 10 million generations sampled every 100 generations with a burn-in of 25,000 (25%), resulted in 150,000 trees overall^13,16^. The S-, M- and L-genomic segments were treated separately in the phylogenetic analyses. Topologies were evaluated by bootstrap analysis of 1,000 iterations, and posterior node probabilities were based on 10 million generations and estimated sample sizes over 100 (implemented in MrBayes)^16^. With a robust phylogeny of shrew-, mole-, bat- and rodent-borne hantaviruses^10^, we readdressed the co-evolutionary relationship between hantaviruses and their hosts that formed the basis of our predictive paradigm for hantavirus discovery, by comparing the degree of concordance between reservoir host and hantavirus cladograms in TreeMap 3β1243^51^.

### Host identification

Total DNA was extracted from lung tissues, using MagDEA DNA 200 Kit (Precision System Science), and PCR amplification of the 1,140-nucleotide Cyt *b* gene and 1,545-nucleotide COI gene^52,53^ was performed with newly designed primer sets: Cy-14724F (5′–GACYARTRRCATGAAAAAYCAYCGTTGT–3′)/Cy-15909R (5′–CYYCWTYIYTGGTTTACAAGACYAG–3′)^16^ and KOD multi enzyme (Toyobo, Osaka, Japan), and MammMt-5533F (5′–CYCTGTSYTTRRATTTACAGTYYAA–3′)/MammMt-7159R (5′–GRGGTTCRAWWCCTYCCTYTCTT–3′) and Phusion enzyme (New England Biolabs, Ipswitch, MA, USA), respectively. Initial denaturation at 95 °C for 2 min was followed by two cycles each of denaturation at 95 °C for 15 s, two-degree step-down annealing from 60 °C to 50 °C for 30 s, and elongation at 68 °C for 1 min 30 s, then 30 cycles of denaturation at 95 °C for 15 s, annealing at 55 °C for 30 s, and elongation at 68 °C for 1 min 30 s, in a Veriti thermal cycler^13^. PCR products were purified by Mobispin S-400 (Molecular Biotechnology, Lotzzestrasse, Germany) and were sequenced directly.

### GenBank accession numbers

MG663534, MG663535 and MG663536 for Đakrông virus; MG524933–MG524935 and LC406452–LC406456 for cytochrome *b* and LC406430–LC406448 for cytochrome c oxidase I of *Aselliscus stoliczkanus* and *Aselliscus dongbacana*.

## Supplementary information

Đakrông virus, a novel mobatvirus (*Hantaviridae*) harbored by the Stoliczka’s Asian trident bat (*Aselliscus stoliczkanus*) in Vietnam
