# Author Correction: Đakrông virus, a novel mobatvirus (*Hantaviridae*) harbored by the Stoliczka’s Asian trident bat (*Aselliscus stoliczkanus*) in Vietnam

**DOI:** 10.1038/s41598-020-57832-y

**Published:** 2020-01-15

**Authors:** Satoru Arai, Keita Aoki, Nguyễn Trường Sơn, Vương Tân Tú, Fuka Kikuchi, Gohta Kinoshita, Dai Fukui, Hoàng Trung Thành, Se Hun Gu, Yasuhiro Yoshikawa, Keiko Tanaka-Taya, Shigeru Morikawa, Richard Yanagihara, Kazunori Oishi

**Affiliations:** 1 Infectious Disease Surveillance Center, National Institute of Infectious Diseases, Tokyo, 162-8640 Japan; 2 Tokyo University of Science, Tokyo, 162-8601 Japan; 3 Institute of Ecology and Biological Resources, Vietnam Academy of Science and Technology, Hanoi, Vietnam; 4 Graduate University of Science and Technology, Vietnam Academy of Science and Technology, Hanoi, Vietnam; 5 Kyoto University Graduate School of Agriculture, Kyoto, 606-8502 Japan; 6 The University of Tokyo Hokkaido Forests, Graduate School of Agricultural and Life Sciences, The University of Tokyo, Furano, Hokkaido, 079-1561 Japan; 7 Faculty of Biology, University of Science, Vietnam National University, Hanoi, Vietnam; 8 Pacific Center for Emerging Infectious Diseases Research, John A. Burns School of Medicine, University of Hawaii at Manoa, Honolulu, HI 96813 USA; 9 Chiba Institute of Science, Chiba, 388-0025 Japan; 10 Department of Veterinary Science, National Institute of Infectious Diseases, Tokyo, 162-8640 Japan

Correction to: *Scientific Reports* 10.1038/s41598-019-46697-5, published online 15 July 2019

The Acknowledgements section in this Article is incomplete.

“We thank Hitoshi Suzuki, Shinichiro Kawada, Satoshi D. Ohdachi and Kimiyuki Tsuchiya for supporting field investigations and for valuable advice. This work was supported in part by a grant-in-aid Research on Emerging and Re-emerging Infectious Diseases, Health Labour Sciences Research Grant in Japan (H25-Shinko-Ippan-008), grants-in-aid on Research Program on Emerging and Re-emerging Infectious Diseases, Japan Agency for Medical Research and Development (AMED) JP15fk0108005, JP16fk0108117, JP17fk0108217, JP18fk0108017 and JP19fk0108097, grant-in aid from the Japan Society for the Promotion of Science 24405045 (Scientific Research grant B), grant-in-aid on NAFOSTED (106-NN.05-2016.14), and VAST-JSPS (QTJP01.02/18-20).”

should read:

“We thank Hitoshi Suzuki, Shinichiro Kawada, Satoshi D. Ohdachi and Kimiyuki Tsuchiya for supporting field investigations and for valuable advice. This work was supported in part by a grant-in-aid Research on Emerging and Re-emerging Infectious Diseases, Health Labour Sciences Research Grant in Japan (H25-Shinko-Ippan-008), grants-in-aid on Research Program on Emerging and Re-emerging Infectious Diseases, Japan Agency for Medical Research and Development (AMED) JP15fk0108005, JP16fk0108117, JP17fk0108217, JP18fk0108017 and JP19fk0108097, grant-in aid from the Japan Society for the Promotion of Science 24405045 (Scientific Research grant B), grant-in-aid on NAFOSTED (106-NN.05-2016.14), and VAST-JSPS (QTJP01.02/18-20). Support was also provided by U.S. Public Health Service grant P30GM114737 from the National Institutes of Health.”

Additionally, this Article contains errors in Reference 38 which was incorrectly given as:

Sikes, R. S. & Animal, C. & Use Committee of the American Society of, M. 2016 Guidelines of the American Society of Mammalogists for the use of wild mammals in research and education. J Mammal 97, 663–688, 10.1093/jmammal/gyw078 (2016).

The correct reference is listed below:

Sikes, R. S. & Animal Care & Use Committee of the American Society of Mammalogists. 2016 Guidelines of the American Society of Mammalogists for the use of wild mammals in research and education. J Mammal 97, 663–688, 10.1093/jmammal/gyw078 (2016).

