# Supplementary material for: Đakrông virus, a novel mobatvirus (Hantaviridae) harbored by the Stoliczka’s Asian trident bat (Aselliscus stoliczkanus) in Vietnam
Source: Sci Rep. 2019 Jul 15;9:10239. doi: 10.1038/s41598-019-46697-5 (PMC6629698; doi:10.1038/s41598-019-46697-5)
Supplement: Supplementary file 1 — Đakrông virus, a novel mobatvirus (Hantaviridae) harbored by the Stoliczka’s Asian trident bat (Aselliscus stoliczkanus) in Vietnam [file 41598_2019_46697_MOESM1_ESM.pdf]

Supplementary information

Đakrông virus, a novel mobatvirus (*Hantaviridae*) harbored by the Stoliczka's Asian trident bat (*Aselliscus stoliczkanus*) in Vietnam

Author information

Satoru Arai[1], Keita Aoki[1,2], Nguyễn Trường Sơn[3,4], Vương Tân Tú[3,4], Fuka Kikuchi[1,2], Gohta Kinoshita[5], Dai Fukui[6], Hoàng Trung Thành[7], Se Hun Gu[8], Yasuhiro Yoshikawa[9], Keiko Tanaka-Taya[1], Shigeru Morikawa[10], Richard Yanagihara[8], Kazunori Oishi[1]

[1] Infectious Disease Surveillance Center, National Institute of Infectious Diseases, Tokyo 162-8640, Japan

[2] Tokyo University of Science, Tokyo 162-8601, Japan

[3] Institute of Ecology and Biological Resources, Vietnam Academy of Science and Technology, Hanoi, Vietnam

[4] Graduate University of Science and Technology, Vietnam Academy of Science and Technology, Hanoi, Vietnam

[5] Kyoto University Graduate School of Agriculture, Kyoto 606-8502, Japan

[6] The University of Tokyo Hokkaido Forests, Graduate School of Agricultural and Life Sciences, The University of Tokyo, Furano, Hokkaido 079-1561, Japan

[7] Faculty of Biology, University of Science, Vietnam National University, Hanoi, Vietnam

[8] Pacific Center for Emerging Infectious Diseases Research, John A. Burns School of Medicine, University of Hawaii at Manoa, Honolulu, HI 96813, USA

[9] Chiba Institute of Science, Chiba 388-0025, Japan

[10] Department of Veterinary Science, National Institute of Infectious Diseases, Tokyo 162-8640, Japan

Supplemental Table 1. Bat samples analyzed for hantavirus RNA

		2012	2013					2014			Total
		Location									
Family	Species	Xuan Lien Nature Reserve	Bac Huong Hoa Nature Reserve	Cu Chi Tunnel	Dakrong Nature Reserve	Khau Ca Nature Reserve	Dong Nai Culture and Nature Reserve	Hoang Lien National Park	Nahang Nature Reserve	Ngoc Son Nature Reserve	
Hipposideridae			20		10	7		3	15	3	53
	Aselliscus dongbacana					2			5		7
	Aselliscus stoliczkanus		10		2						12
	Hipposideros armiger		1		1			3	2		7
	Hipposideros cf. larvatus 1		3		3						6
	Hipposideros cf. larvatus 2					3			3		6
	Hipposideros cf. larvatus 3		1								1
	Hipposideros pomona 1					2			3	3	8
	Hipposideros pomona 2				3						3
	Hipposideros pratti		3								3
Megadermatidae			1								1
	Megaderma spasma		1								1
Pteropodidae		2				2	6				10
	Cynopterus cf. branchyotis						3				3
	Cynopterus cf. sphinx	2				2					4
	Cynopterus horsfieldi						1				1
	Megaerops niphanae						2				2
Rhinolophidae		8	15	1	16	10	7	16	9		82
	Rhinolophus acuminatus						3				3
	Rhinolophus affinis	3	3		3	1		3			13
	Rhinolophus cf. macrotis							1			1
	Rhinolophus cf. pusillus				1			3	2		6
	Rhinolophus cf. siamensis	1	1		1	1		2	1		7
	Rhinolophus cf. thomasi					2		3	1		6
	Rhinolophus luctus						1				1
	Rhinolophus malayanus		3		3						6
	Rhinolophus microglobosus	2			2	1			3		8
	Rhinolophus monoceros		3								3
	Rhinolophus paradoxolophus		1			2			1		4
	Rhinolophus pearsonii 1	2									2
	Rhinolophus pearsonii 2		1		3						4
	Rhinolophus pearsonii 3							1			1
	Rhinolophus pearsonii 4					3		2	1		6
	Rhinolophus sinicus		3	1	3		3	1			11
Vespertilionidae		4	8		5	5	17	17	13		69
	Harpiola isodon							1			1
	Hypsugo cadornae					1					1
	Kerivoula cf. papillosa						3				3
	Kerivoula cf. titania		1						3		4
	Kerivoula depressa		1								1
	Kerivoula dongduongana		1			1					2
	Kerivoula furva					1		1			2
	Kerivoula hardwickii						2				2
	Kerivoula kachinensis								3		3
	Miniopterus magnater		3								3
	Murina sp.							2			2
	Murina cf. huttoni							3			3
	Murina cyclotis				2						2
	Murina feae		1						1		2
	Murina tubinaris					1			3		4
	Myotis annamiticus		1		3						4
	Myotis annectans					1					1
	Myotis cf. montivagus							1			1
	Myotis laniger	3						3	2		8
	Myotis muricola	1					3				4
	Myotis rosetti						2				2
	Phoniscus jagorii						1		1		2
	Plecotus homochrous							4			4
	Scotomanes ornatus							2			2
	Tylonycteris fulvidus						3				3
	Tylonycteris pachypus						3				3
	Total	14	44	1	31	24	30	36	37	3	215

Supplemental table 2
Oligonucleotide primers for amplification of the full-length S, M and L segments of DKGV strain 2913B72.

	Primer name	Sequences (5'-3')	Polarity
S segment	HTS-3R	TAGTAGTAIGCTCCYT	-
	S-437F	SWGGTCARACTGCHRAYTGG	+
	Cro2R(1126R)	AIGAYTGRTARAAIGAIGAYTTYTT	-
	Cro2F(685F)	AGYCCIGTIATGRGWGTIRTYGG	+
	XSV-S6R	AGITCIGGRTCCATRTCRTCICC	-
	DGS-453R	GTARAAGGRAATGTCASCAGGT	-
	DGS-596F	TGTGTCACTTCCTACTGGTCAG	+
	DGS-704R	GAGCCTTAGTCTCWGCAGCRT	-
	XSS-729R	CCWATIACYCCCATKACWGGRCT	-
	SMGS-1079F	ATIATGGCWTCTAAGCTTGTYGG	+
M segment	XM-3endR	TAGTAGTAKRCTCCGCARGA	-
	MKWM-917R	TRTCATGATCTTCICCATRTGG	-
	T-M1199F	TAAVTTCAMCAACATGTCT	+
	T-M1485R	CCAGCCAAARCARAATGT	-
	HTM-1490F	TGTGTICCWGGITTYCATGGIT	+
	XSM-1973F	CAKTAATATGGGCWKCTAGTGCA	+
	XDM-2017R	ACICCRTGWGCTGTRTCYTGCCA	-
	HTM-2409R	CCACAIGCWGTRCAICCWGT	-
	MKWM-2631R	CATGATRTCICCAGGRTCICC	-
	XDM-2841F	TIATGTGGKCTGAYCCWGATGG	+
	XSM-2959R	CTGAACCCCAWGMICCTTCAAT	-
	XDM-3360F	GKWTRTTYCAYGGMAACTGGTGG	+
L segment	HL-3endR	TAGTAGTAKRCTCCGGA	-
	PHL-173F	GATWAAGCATGAYTGGTCTGA	+
	XAL-948F	CAATMTGAGTATTCMCCWKCTAC	+
	XAL-1534F	CAAARTWYTGGTCTGTYCATGC	+
	XAL-2137F	AGGWGCIAGTGGWGTKTATCC	+
	XSL-2227R	TGCTTCTTCTGTCATWGTICCAYG	-
	HAN-L-F1	ATGTAYGTBAGTGCWGATGC	+
	HAN-L-R1	AACCADTCWGTYCCRTCATC	-
	HAN-L-F2	TGCWGATGCHACIAARTGGTC	+
	HAN-L-R2	GCRTCRTCWGARTGRTGDGCAA	-
	DGL-3225F	TACGTGGIAATTGGTTGCARGG	+
	XSL-3719F	TRGCTGCTKCWCARASTMGKTGTG	+
	PHL-5167R	CATAYTGYTTHCCTGAATAWGC	-
	HTL-5278F	GTGCAAGSYTAGARATITYYTGGG	+
	XDL-5809R	GCAYTAGGRGGRATWGATGCAGG	-
	XDL-6088R	GTAGRAAATGCTCWATGTCATC	-

Mixed bases: B= G, T, C; D= G, A, T; H= A, T, C; K= G, T; M= A, C; N= A, T, G, C; R = A, G; W= A, T; and Y= C, T.

Supplemental table 3. Gene accession numbers of cytochrome b (Cyt*b*) and relataed hantaviruses.

Order	Family	Host species of virus	Cyt*b*	Virus abbreviation	Clone	S segment	M segment	L segment
Chiroptera	Hipposideridae	*Aselliscus stoliczkanus*	KU161570	DKGV	VN2913B72	MG663534	MG663535	MG663536
Chiroptera	Hipposideridae	*Hipposideros pomona*	JX912954	XSV	VN1982B4	KC688335	KU976427	JX912953
Chiroptera	Hipposideridae	*Hipposideros ruber*	EU934474	MAKV	GB303	–	–	KT316176
Chiroptera	Emballonuridae	*Taphozous melanopogon*	LC406449	LAIV	BT20	KM102247	KM102248	KM102249
Chiroptera	Pteropodidae	*Rousettus amplexicaudatus*	KU950716	QZNV	MT1720/1657	KU950713	KU950714	KU950715
Chiroptera	Rhinolophidae	*Rhinolophus affinis*	JX465358	LQUV	Ra-25	JX465415	JX465397	JX465381
Chiroptera	Vespertilionidae	*Nyctalus noctula*	JX570902	BRNV	7/2012/CZE	KX845678	KX845679	KX845680
Chiroptera	Vespertilionidae	*Neoromicia nanus*	EU797428	MOYV	KB576	–	–	KJ000540
Chiroptera	Vespertilionidae	*Pipistrellus abramus*	JX465352	HUPV	Pa-1	JX473273	–	JX465369
Chiroptera	Nycteridae	*Nycteris hispida*	HQ693722	MGBV	1209	–	–	JN037851
Eulipotyphla	Soricidae	*Myosorex geata*	JX193701	ULUV	FMNH158302	JX193695	JX193696	JX193697
Eulipotyphla	Soricidae	*Myosorex zinki*	JX193702	KMJV	FMNH174124	JX193698	JX193699	JX193700
Eulipotyphla	Soricidae	*Suncus murinus*	DQ630386	TPMV	VRC66412	AY526097	EU001329	EU001330
Eulipotyphla	Soricidae	*Crocidura lasiura*	KJ004674	MJNV	Cl05-11	EF641804	EF641798	EF641806
Eulipotyphla	Soricidae	*Crocidura shantungensis*	HQ663932	JJUV	SH42	HQ663933	HQ663934	HQ663935
Eulipotyphla	Soricidae	*Crocidura obscurior*	KC684096	AZGV	KBM15	JF276226	JF276227	JF276228
Eulipotyphla	Soricidae	*Crocidura theresae*	DQ521043	TGNV	Tan826	EF050455	–	EF050454
Eulipotyphla	Soricidae	*Crocidura douceti*	KC684929	BOWV	VN1512	KC631782	KC631783	KC631784
Eulipotyphla	Soricidae	*Anourosorex squamipe*	AB175090	CBNV	CBN3	EF543524	EF543526	EF543525
Eulipotyphla	Soricidae	*Sorex caecutiens*	KF974362	ARTV	MukawaAH301	KF974360	KF974359	KF974361
Eulipotyphla	Soricidae	*Sorex roboratus*	AB175128	KKMV	MSB148794	GQ306148	GQ306149	GD306150
Eulipotyphla	Soricidae	*Sorex cinereus*	FJ667512	ARRV	MSB734418	EF650086	–	EF619961
Eulipotyphla	Soricidae	*Sorex monticolus*	FJ667514	JMSV	MSB144475	FJ593499	FJ593500	FJ593501
Eulipotyphla	Soricidae	*Sorex araneus*	FJ667524	SWSV	mp70	EF636024	EF636025	EF636026
Eulipotyphla	Soricidae	*Neomys fodiens*	KC537797	BOGV	2074	–	JX990966	JX990965
Eulipotyphla	Talpidae	*Urotrichus talpoides*	EU918371	ASAV	N10	EU929072	EU929075	EU929078
Eulipotyphla	Talpidae	*Scalopus aquaticus*	HM461914	RKPV	MSB57412	HM015223	HM015222	HM015221
Eulipotyphla	Talpidae	*Talpa europaea*	FJ715340	NVAV	Te34	KR072621	KR072622	KR072623
Eulipotyphla	Talpidae	*Neurotrichus gibbsii*	FJ595237	OXBV	Ng1453	FJ539166	FJ539167	FJ593497
Rodentia	Muridae	*Apodemus agrarius*	AB303225	HTNV	76-118	NC_005218	NC_005219	NC_5222
Rodentia	Muridae	*Apodemus flavicollis*	AY158451	DOBV/BGDV	Greece	NC_005233	NC_005234	NC_005235
Rodentia	Muridae	*Apodemus peninsulae*	AB073811	SOOV	SOO-1	AY675349	AY675353	DQ056292
Rodentia	Muridae	*Rattus norvegicus*	KY356141	SEOV	HR80-39	NC_005236	NC_005237	NC_005238
Rodentia	Muridae	*Hylomyscus simus*	DQ212188	SANGV	SA14	JQ082300	JQ082301	JQ082302
Rodentia	Muridae	*Stenocephalemys albipes*	AF518346	TIGV	ET2121	KU934010	KU934009	KU934008
Rodentia	Cricetidae	*Myodes glareolus*	JX477304	PUUV	Sotkamo	NC_005224	NC_005223	NC_005225
Rodentia	Cricetidae	*Myodes regulus*	NC_016427	MUJV	11-1	JX028273	JX028272	JX028271
Rodentia	Cricetidae	*Myodes rufocanus*	LC406450	HOKV	Kitahiyama	AB675463	AB676848	AB712372
Rodentia	Cricetidae	*Microtus pennsylvanicus*	AF119279	PHV	PH-1	Z49098	X55129	EF646763
Rodentia	Cricetidae	*Microtus arvalis*	AY220770	TULV	M5320v	NC_005227	NC_005228	NC_005226
Rodentia	Cricetidae	*Oligoryzomys longicaudatus*	AF346566	ANDV	Chile9717869	NC_003466	NC_003467	NC_003468
Rodentia	Cricetidae	*Peromyscus maniculatus*	AF119261	SNV	NMH10	NC_005216	NC_005215	NC_005217
Macroscelidea	Macroscelididae	*Elephantulus edwardii**	DQ901019					
Macroscelidea	Macroscelididae	*Elephantulus intufi**	DQ901206					
Macroscelidea	Macroscelididae	*Elephantulus rupestris**	DQ901201					
Chiroptera	Hipposideridae	*Hipposideros cineraceus***	KX458067					
Chiroptera	Hipposideridae	*Coelops frithi***	DQ888674					

– indicates that no sequences are available.
* Outgroup for writing host phylogenetic tree.
* Supplemental host for writing host phylogenetic tree.
Abbreviations: ANDV, Andes virus; ARTV, Artybash virus; ARRV, Ash River virus; ASAV, Asama virus; AZGV, Azagny virus; BOWV, Bowé virus; BRNV, Brno virus; CBNV, Cao B ằng virus; DKGV, Dakrong virus; DOBV/BGDV, Dobrava-Belgrade virus; HTNV, Hantaan virus; HOKV, Hokkaido virus; HUPV, Huángpí virus; JJUV, Jeju virus; JMSV, Jemez Springs virus; KKMV, Kenkeme virus; KMJV, Kilimanjaro virus; LAIV, Láibīn virus; LQUV, Lóngquán virus; MAKV, Makokou virus; MGBV, Magboi virus; MJNV, Imjin virus; MOYV, Mouyassué virus; MUJV, Muju virus; NVAV, Nova virus; OXBV, Oxbow virus; PHV, Prospect Hill virus; PUUV, Puumala virus; QZNV, Quezon virus; RKPV, Rockport virus; SANGV, Sangassou virus; SEOV, Seoul virus; SNV, Sin Nombre virus; SOOV, Soochong virus; SWSV, Seewis virus; TGNV, Tanganya virus; TIGV, Tigray virus; TPMV, Thottapalayam virus; TULV, Tula virus; ULUV; Uluguru virus; XSV, Xuân Sơn virus; YKSV, Yákèshí virus.
